# mTOR-Related Brain Dysfunctions in Neuropsychiatric Disorders

**DOI:** 10.3390/ijms19082226

**Published:** 2018-07-30

**Authors:** Larisa Ryskalin, Fiona Limanaqi, Alessandro Frati, Carla L. Busceti, Francesco Fornai

**Affiliations:** 1 Human Anatomy, Department of Translational Research and New Technologies in Medicine and Surgery, University of Pisa, Via Roma 55, 56126 Pisa, Italy; larisa.ryskalin@unipi.it (L.R.); f.limanaqi@studenti.unipi.it (F.L.); 2 I.R.C.C.S. Neuromed, Via Atinense 18, 86077 Isernia, Italy; alessandro.frati@uniroma1.it (A.F.); clbusceti@libero.it (C.L.B.)

**Keywords:** mTOR, rapamycin, autophagy, protein aggregation, methamphetamine, schizophrenia

## Abstract

The mammalian target of rapamycin (mTOR) is an ubiquitously expressed serine-threonine kinase, which senses and integrates several intracellular and environmental cues to orchestrate major processes such as cell growth and metabolism. Altered mTOR signalling is associated with brain malformation and neurological disorders. Emerging evidence indicates that even subtle defects in the mTOR pathway may produce severe effects, which are evident as neurological and psychiatric disorders. On the other hand, administration of mTOR inhibitors may be beneficial for a variety of neuropsychiatric alterations encompassing neurodegeneration, brain tumors, brain ischemia, epilepsy, autism, mood disorders, drugs of abuse, and schizophrenia. mTOR has been widely implicated in synaptic plasticity and autophagy activation. This review addresses the role of mTOR-dependent autophagy dysfunction in a variety of neuropsychiatric disorders, to focus mainly on psychiatric syndromes including schizophrenia and drug addiction. For instance, amphetamines-induced addiction fairly overlaps with some neuropsychiatric disorders including neurodegeneration and schizophrenia. For this reason, in the present review, a special emphasis is placed on the role of mTOR on methamphetamine-induced brain alterations.

## 1. Introduction

**Figure 1:**
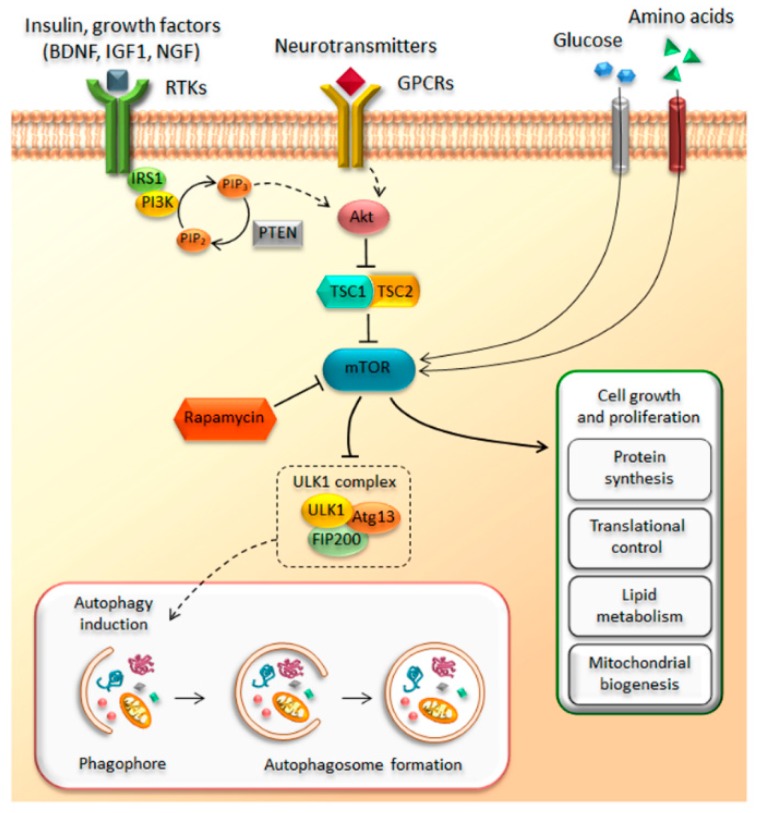
The mammalian target of rapamycin (mTOR) pathway. The cartoon summarizes the main up- and down-stream components of the mTOR pathway. Growth factors, glucose, and amino acids activate mTOR, which in turn promotes protein synthesis, lipid metabolism, and mitochondrial biogenesis, while autophagy is under the negative control mTOR. GPCRs—G-protein coupled receptors. TSC—tuberous sclerosis complex; TSC1—hamartin; TSC2—tuberin; RTKs—receptor tyrosine kinase receptors; Akt—protein kinase B; PTEN—Phosphatase and Tensin Homolog; BDNF—Brain-derived neurotrophic factor; IGF—insulin-like growth factor; NGF—nerve growth factor.

The discovery of the mammalian target of rapamycin (mTOR) dates back to early 1970s with the collection of a soil sample of Easter Island (Rapa Nui) and the serendipitous identification of a lipophilic macrolide produced by the soil bacterium *Streptomyces hygroscopicus* [1]. This natural compound called rapamycin was initially developed as an antifungal drug, but it soon raised considerable interest because of its unexpected and, at that time, undesired immunosuppressive side effects. The discovery of rapamycin-mediated anti-proliferative effects on immune cells was a milestone in organ transplantation [2,3,4,5,6]. However, the finding of anti-proliferative activities, way beyond the immunosuppressive properties, disclosed novel potential therapeutic uses that fueled research on its mechanisms of action [7,8,9,10]. Nowadays, it is well established that rapamycin exerts its effects by forming a complex with FK506-binding protein 12 (FKBP12), which in turn inhibits the target of rapamycin (TOR). TOR is a large (289 kDa), evolutionarily highly conserved, serine/threonine kinase, which represents the catalytic domain of a multiprotein complex named TORC (target of rapamycin complex) [5,11,12,13]. The need of FKBP-12 to mediate the effects of rapamycin on TORC is demonstrated by a lack of effects induced by rapamycin when the binding between FKBP12 and rapamycin is occluded by a missense point mutations within the FRB domain of TOR [14,15]. In mammals, TOR kinase, also known as mTOR (i.e., mammalian TOR), is ubiquitously expressed in all cell types and it assembles with several scaffolds and regulatory subunits to form two distinct multiprotein complexes, hereinafter referred as mTOR complex 1 (mTORC1) and mTOR complex 2 (mTORC2) [16,17,18,19]. These mTOR complexes share four components that are identical; they possess (i) a catalytic subunit, along with (ii) a small protein known as mLSt8, which represent the core of both complexes. These in turn are composed of two more components, namely (iii) the Tti1/Tel2 associated regulatory proteins, which create a scaffold for recruitment of substrates; and (iv) the negative regulator Deptor, which inhibits the substrate binding [20,21,22]. In addition, there are specific subunits depending on which mTOR complex is considered. In detail, mTORC1 contains the scaffold protein Raptor and the inhibitory subunit PRAS40 as key components, while mTORC2 specifically associates with the regulatory subunit Protor 1/2 and scaffold proteins Rictor and mSIN1, which help the complex assembly [20,23,24,25,26,27,28]. The mTOR complex represents a downstream substrate of the PI3K/PTEN/Akt pathway, which controls cell growth, proliferation, metabolism, and motility in response to bioenergetics and nutritional requests [29,30]. Extracellular and environmental stimuli are conveyed through mTOR via the PI3K/PTEN/Akt pathway. The binding of insulin and growth factors to tyrosine kinase receptors (RTKs) activates the lipid kinase PI3K, which phosphorylates phosphatidylinositol-4,5-phosphate (PIP2) to generate phosphatidylinositol-3,4,5-phosphate (PIP3). This second messenger recruits Akt, which promotes mTOR activity. This occurs via the phosphorylation of the tuberous sclerosis complex (TSC), which impairs its inhibitory activity on mTOR. TSC is a heterodimer that is composed of hamartin (TSC1) and tuberin (TSC2). In this way, the activation of PI3K/Akt signaling leads to the activation of mTOR through TSC inhibition. Once activated, mTOR promotes various activities including protein synthesis, ribosome, and lipid biogenesis [21,31] (Figure 1). Among these activities, mTOR inhibits autophagy [32]. In mammalian cells, three main autophagy pathways are described, all providing the lysosomal degradation of intracellular components: (i) micro-autophagy; (ii) macro-autophagy; and (iii) chaperone-mediated autophagy (CMA). In detail, micro-autophagy enwraps small portions of cytosol and proteins into lysosomes [33], while macro-autophagy sequesters “in bulk” cytosolic cargoes, including organelles, within autophagosomes to merge with lysosomes [34]. Finally, CMA is a rather selective process where proteins are bound to cytosolic chaperones (e.g., LAMP-2) to be recognized and translocated across the lysosomal membrane for degradation [35]. In the present review, we focus on macro-autophagy (hereinafter referred to as autophagy), which is mostly related to mTOR activity. In detail, mTOR inhibits autophagy by suppressing the ULK1 complex, which consists of several autophagy-related proteins (ULK1, Atg13, FIP200). In fact, by phosphorylating the ULK1 complex, mTOR inhibits early steps in the biogenesis of autophagosomes [36,37,38,39] (Figure 1). Conversely, rapamycin-induced mTOR inhibition strongly activates autophagy. In eukaryotic cells, autophagy represents the main cell clearing system. The autophagy pathway is initiated through a nascent double-layered membrane vacuole, which, at early stages, is not yet complete and is named phagophore. The maturation of the phagophore leads to seal the vacuole, which is then named autophagosome. At this stage, the vacuole stains for beclin-1 (the ortholog of yeast Atg6) and LC3 (Atg8), which are thus considered gold standard autophagy markers [40]. The autophagosome carries a variety of substrates to the lysosomal compartment, which possesses a rich enzymatic activity. In detail, when the autophagosome merges with the lysosome, the catalytic organelle autophagolysosome is generated, where degradation and recycling of sequestered cytosolic cargoes occurs. Autophagy can be tuned very finely to obtain either slight or robust effects based upon specific cell needs. For instance, autophagy is strongly induced by nutrient depletion, which occurs during cell starvation. In these conditions, enhanced protein degradation within lysosomes results in the generation of single amino acids. This is the prototype for extreme cell conditions when cell survival is jeopardized; however, a slight autophagy activation is needed in baseline conditions to keep steady the level of misfolded proteins that naturally occur within a living cell. An appropriate tuning of autophagy avoids the burden of aged structures within the cell. Along with degrading misfolded proteins, autophagy degrades altered subcellular organelles (mitochondria, endoplasmic reticulum, ribosomes, and even synaptic vesicles). Thus, when a mild failure in the autophagy pathway occurs, the cell still survives, though such a decreased protein turnover during prolonged time intervals is detrimental. In fact, this may alter a variety of cell activities and may even produce toxicity. This is why it takes time to appreciate the effects of a slight relenting of protein and organelle turnover due to a mild autophagy deficiency. In fact, in these conditions, the cell may easily cope with moderate energy demands. In contrast, when the production of altered structures exceeds a reduced autophagy activity, these accumulate and become visible over the years through intracellular deposits that contain altered protein aggregates. This is probably why an autophagy defect eventually leads to slowly developing neuronal inclusions. This is facilitated by the inner nature of specific proteins such as alpha synuclein, which is prone to aggregate because 30% of its native form undergoes spontaneous oligomerization independently of the metabolic conditions [41]. Thus, autophagy defects are expected to generate protein aggregates, which in turn promote toxicity and cell death [42]. This is kind of an oversimplification, but it helps to schematize the concept of autophagy as a cell clearing system beyond its powerful energetic effects. Unlike most cell types, neurons are extremely vulnerable to autophagy impairment. This is not surprising when considering that adult neuronal cells are post-mitotic. Therefore, neurons cannot profit from mitosis to dilute potential toxic waste within daughter cells. In fact, mice deficient for autophagy-related proteins, such as Atg5 or Atg7, show inclusion bodies and marked neuronal loss [43,44]. In line with this, mTOR-dependent impairment of autophagy is implicated in various neuropsychiatric disorders such as dementia, movement disorders, motor neuron disease, seizures, brain ischemia, autism, affective disorders, addiction, and schizophrenia [45,46,47,48,49,50,51,52,53,54,55,56,57]. While the involvement of autophagy in neurological disorders is intensely investigated, the evidence about an involvement of autophagy in psychiatric disorders is less clear. Therefore, the aim of the present manuscript is to mention the role of mTOR-dependent autophagy in neurodegeneration while emphasizing its role in methamphetamine (METH) addiction and psychiatric disorders, namely schizophrenia. Remarkably, a growing evidence shows that mTOR dysfunction may underlie a variety of psychiatric syndromes, including mood disorders, drug addiction, and schizophrenia. In fact, many psychotropic drugs including mood stabilizers and neuroleptics are powerful autophagy inducers [58,59,60,61,62,63,64,65,66,67]. In such an attempt, we hint to the role of mTOR-dependent autophagy as a hub in etiologically distinct brain disorders from neurodegeneration to METH abuse and schizophrenia.

## 2. A Short Overview on Autophagy Impairment in Neurodegenerative Disorders

Dysfunctional autophagy appears as a recurring feature in neurodegenerative disorders (NDDs), such as Parkinson’s disease (PD) and Alzheimer’s disease (AD), where a defect along the autophagy pathway occurs at different stages [68,69,70,71,72,73,74,75,76]. The accumulation of aggregate-prone proteins triggers the formation of cytoplasmic and/or extracellular neuronal inclusions within specific brain areas. This occurs in PD, where aggregate-prone alpha synuclein accumulates in the so-called Lewy bodies, which are mostly found within spared dopaminergic (DA) neurons of the substantia nigra pars compacta (SNpc) [77,78], as well as within extra-nigral neuronal populations [79]. Remarkably, genetic ablation of Atg7 specifically within dopamine (DA) neurons fully reproduces PD pathology, including the formation of Lewy bodies, which stain for alpha synuclein, pointing at a key role for autophagy in DA-related disorders [80]. Likewise, AD cortical pathology features abnormal intracellular hyperphosporylated tau protein, which forms fibrils known as neurofibrillary tangles (NFT) along with extracellular amyloid-β (Aβ) plaques [81]. Pathological TAR DNA-binding protein 43 (TDP-43) is a major component of inclusions that are found in most cases of amyotrophic lateral sclerosis (ALS) and in frontotemporal lobar degeneration (FTLD) [82,83]. Remarkably, further investigation on the autophagy pathway revealed that all these misfolded proteins are autophagy substrates depending on mTOR activity [84,85,86,87,88]. In an attempt to attribute a specific protein accumulation to the onset of a specific disorder within the aim of describing a sort of “precision medicine”, investigators faced the hurdle that these protein aggregates may indeed be shared by different disorders and different proteins may aggregate in the same disease. In fact, TDP-43 positive inclusions are found within the very same neurons containing tau-positive NFTs and alpha synuclein-positive Lewy bodies of post-mortem brains from patients with AD and dementia with Lewy bodies (DLB), respectively [89]. In addition, most DLB patients show most features of AD (i.e., hyperphosphorylated tau deposits and Aβ) to various extents [90]. Moreover, alpha synuclein immunoreactivity often co-localizes within huntingtin polyglutamine-positive aggregates in brain sections from patients with late-stage Huntington’s disease (HD) patients [91], or even within SOD1-positive inclusions, as revealed by immunohistochemical analysis performed in post-mortem brain and spinal cord from cases of familial ALS [92]. All this evidence challenges the concept of a protein-specific vulnerability to characterize each disease leading to clinically distinct neurological phenotypes. Such a contamination even overcomes the clear cut between neurodegenerative disorders and acute cerebral ischemia/chronic hypoperfusion [93]. A failure in autophagy-dependent handling of misfolded proteins impedes the clearance of these substrates that are likely to accumulate within the cell. Therefore, a common pathogenesis underlying all these NDDs disorders has been linked to autophagy inhibition due to mTOR hyperactivation [52,54,94,95,96,97]. For instance, an increased mTOR activity correlates with accumulation of Aβ and hyperphosphorylated tau in AD brains [98,99]. On the other hand, some evidence indicates that suppressing mTOR activity ameliorates AD cognitive defects by decreasing Aβ and tau pathology [100]. Again, rapamycin and rapalogs protect against toxicity produced by a number of misfolded proteins encompassing alpha synuclein, TDP43, and hyperphosphorylated tau [101,102,103,104]. Therefore, mTOR inhibitors, as such autophagy inducers, may be useful to boost relented cell clearing mechanisms by decreasing abnormal aggregate-prone proteins, which is supposed to ameliorate neurodegeneration.

## 3. Beyond Classic Neurodegeneration

The beneficial effects of mTOR inhibitors have been demonstrated in patients affected by neurodevelopmental disorders [105,106,107,108]. In fact, compounds belonging to the macrolides family, such as rapamycin and rapalogs, and ameliorate cognitive, affective, and overall psychiatric symptoms, which is in line with an Akt–mTOR-dependent antidepressant and mood stabilizing effect [105,109,110]. This is further supported by mounting evidence obtained in rodent models, which demonstrate that rapamycin normalizes impaired social interactions and reverses behavioral defects [61,109,111,112,113,114]. This appears indeed as a continuum rather than a concomitance of different effects, as patients affected by neurodegenerative disorders frequently develop psychiatric symptoms like mood alterations, depression, and schizophrenia, which may appear early during disease development and then may persist throughout the disease course [115,116,117,118,119,120]. As briefly reported, despite a high number of studies correlating mTOR and autophagy with neurological disorders [45,46,47,48,49,50,51,52,53,54,55,56,57], only a few studies addressed such an issue in psychiatric disorders such as schizophrenia [121,122]. This is likely to depend on the lower amount of biological and pathological investigations that are carried out in these patients and the scarce knowledge about the molecular neurobiology of disease-concerning psychiatric disorders. In schizophrenia, a progressive synaptic disorder is likely to promote neurodegeneration [123]. In support of this view, autoptic studies on schizophrenic brains have revealed the presence of neuronal inclusions (see Section 5), which may depend on dysfunctional mTOR-related cell clearing systems. Similarly, neuronal inclusions occur in METH abusers [124], confirming what was previously demonstrated in animal models [125,126]. As detailed in the following paragraph, METH exerts disruptive effects on DA neurotransmission, which translate into abnormal stimulation of post-synaptic DA receptors, mainly D1-type DA receptors (D1R), thus leading to non-canonical signaling cascades sustaining behavioral alterations that overlap with schizophrenia-like symptoms (i.e., visual and auditory hallucinations and delusions) [127,128,129,130]. Increased activity of D1R is considered as a major determinant of neuropsychiatric alterations occurring in both METH models/abusers and in schizophrenia [130,131,132]. This is key, because abnormal stimulation of D1R and subsequent signaling cascades were recently shown to produce an over-activation of mTOR and inhibition of the autophagy machinery [133]. In addition, several susceptibility genes for schizophrenia (e.g., *DISC1*, *NRG1/ErbB4*, and *CRMP2*), which are involved in either pre-synaptic DA release or post-synaptic D1R-related cascades, are similarly dysregulated by METH. Interestingly, they all converge on mTOR signaling (see Section 6, Table 1). In fact, mTOR-induced autophagy inhibition exacerbates the ultrastructural effects of METH [126,134,135,136], while rapamycin administration reverts both behavioral and morphological alterations induced by METH [137]. Such an issue will be further dealt with in the next paragraph. Here, we wish to point out that the detrimental effects of METH on both DA neurotransmission and mTOR-dependent cell clearing systems produce behavioral alterations that are reminiscent of schizophrenia. Thus, METH addicted brains may represent a bridge that connects neurodegenerative and psychiatric disorders. Understanding the molecular and cellular mechanisms that operate during METH toxicity is expected to increase our insight into the neurobiology of schizophrenia. Thus, in the next paragraph we discuss evidence on how altered mTOR and an impaired autophagy pathway may indeed represent a common hub between drug addiction and schizophrenia.

## 4. Bridging Neurodegeneration and Psychiatric Disorders: The Paradigm of Methamphetamine-Addicted Brains

**Figure 2:**
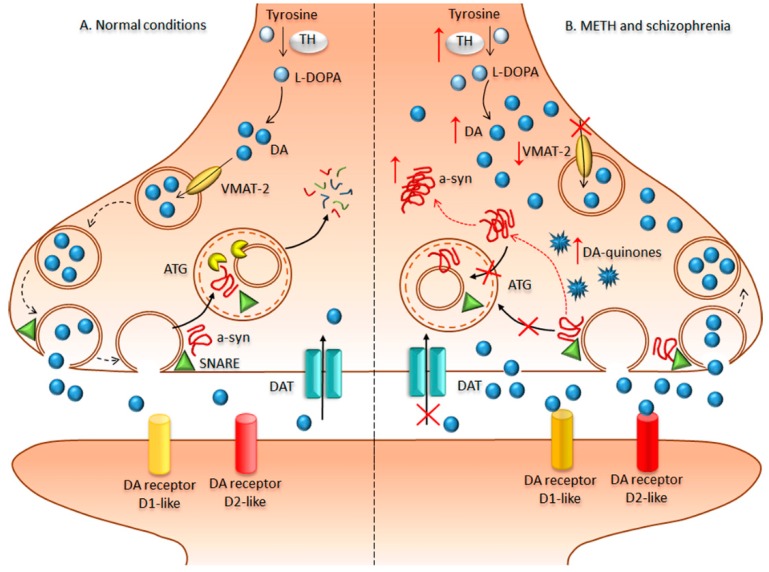
Overlap of dopamine-dependent molecular mechanisms underlying methamphetamine (METH) and schizophrenia. In normal conditions (**A**), the amount of intra-cytosolic dopamine is determined by the rate limiting enzyme tyrosine hydroxylase (TH), which converts tyrosine into L-dihydroxyphenylalanine (L-DOPA) and eventually dopamine (DA). DA is selectively taken-up into synaptic vesicles by the vesicular monoamine transporter type-2 (VMAT-2), which is key to surveil the physiological storage of vesicular DA. DA-containing synaptic vesicles are coated with soluble NSF (N-ethylmaleimide-sensitive factor) attachment protein receptor (SNARE) proteins co-chaperoned by alpha-synuclein, which mediate docking, priming, and release of DA-synaptic vesicles via exocytosis. Once exocytosis has occurred, synaptic vesicles and their associated proteins are endocytosed and sorted for autophagy (ATG) degradation. In this way, ATG monitors the amount of releasable DA synaptic vesicles, thus playing a key role in restraining DA release and in the turnover of synaptic proteins. In the synaptic cleft, the dopamine transporter (DAT) is key to take-up extracellular DA in order to guarantee a physiological stimulation of post-synaptic DA receptors. On the other hand, METH addicted and schizophrenic brains (**B**) feature alterations of DA metabolism and handling, which consist of the following: (i) increased levels of TH, which produces high levels of intra-cytosolic DA; (ii) a decrease in VMAT-2, which leads to a loss of DA vesicular storage and increases the amount freely diffusible intra-cytosolic DA; (iii) free cytosolic DA is highly prone to auto-oxidation into reactive DA-quinones, which produce structural modifications of presynaptic proteins such as alpha synuclein; (iv) a rapid and massive release of DA occurs via either exocytosis or efflux from the axoplasm; (v) extracellular DA rapidly accumulates as DAT is inhibited or downregulated, thus leading to abnormal stimulation of post-synaptic DA receptors, mainly D1-like receptors; (vi) dysfunctions in the ATG machinery, which cannot restrain DA release, are likely to play a key role in such a mechanism. In addition, impaired ATG cannot handle the oxidatively modified alpha-synuclein, thus leading to a progressive accumulation of alpha-synuclein aggregates fueling synaptic pathology.

METH is a widely abused drug that rapidly enters and persists within the central nervous system (CNS), thereby exerting powerful addictive effects [138,139,140]. In humans, the sensitizing effects of prolonged chronic METH intake are considered a major determinant to the occurrence and relapse of psychoses, which mirror those occurring in schizophrenic patients. In fact, METH-addicted patients commonly develop psychoses with positive symptoms similar to those of schizophrenia, which led to the use of METH as an experimental model of schizophrenia (Figure 2). In fact, psychotic patients are oversensitive to amphetamines [141,142]. Accordingly, clinical evidence points towards an elevation of pre-synaptic DA synthesis and release as a key event for schizophrenia [141,143,144]. Likewise, the psychostimulant effects experienced by METH-addicted patients rely on increased DA synthesis and massive DA release from nerve terminals within limbic areas as occurring in the schizophrenic brain [145,146,147,148,149]. Such an abnormal DA release produces peaks of extracellular DA, which cannot be taken up within nerve terminals, because METH inhibits and reverts the direction of the dopamine transporter (DAT). In line with this, recent studies suggest that DAT expression is significantly reduced in the midbrain of postmortem schizophrenic samples [150], which is reminiscent of the METH-addicted brain [151,152]. Upon METH administration, the massive amount of extracellular DA is followed by DA depletion, which translates into a pulsatile stimulation of post-synaptic DA receptors. This triggers non-canonical transduction pathways driving phenotypic changes at the level of post-synaptic neurons. The abnormal activity of DA receptors is an additional commonality between METH and schizophrenia, which is likely to represent the molecular mechanism underlying behavioral alterations [130,131,132]. Remarkably, mTOR over-activation was recently linked to METH-induced behavioral sensitization, while rapamycin prevents such an effect [137]. Likewise, rapamycin was found to be beneficial for ameliorating psychotic symptoms [109,112,114,153,154]. The relevance of autophagy for sustaining these mTOR-induced effects is confirmed by drugs inducing autophagy independently of mTOR activation. In fact, lithium is able to delay METH-induced sensitization, while being a powerful treatment in schizophrenia [155]. Behavioral alterations driven by abnormal DA receptor activity are widely dependent on the amount of DA released from pre-synaptic terminals. Noteworthy, genetic ablation of autophagy was shown to produce an extremely powerful DA release upon electrical stimuli, suggesting that autophagy is key to restrain DA release both upon basal neural activity and mostly after rapamycin-induced autophagy [156]. These findings strongly suggest that an autophagy dysfunction acts both at pre- and post-synaptic level to alter DA neurotransmission during both METH administration and schizophrenia (Figure 2). This confirms our previous studies showing that both genetic and pharmacological autophagy inhibition worsen the effects of METH administration [126]. Given the paucity of studies showing such a role in schizophrenia, dissecting the molecular mechanisms underlying autophagy dysfunction in METH may provide insights in the pathophysiology of schizophrenia. In line with this, METH produces ultrastructural alterations reflecting dysfunctional autophagy flux, which are DA-dependent. In fact, METH produces a massive increase of endogenous intra-cytosolic DA levels by inhibiting and reverting the direction of the vesicular monoamine transporter type 2 (VMAT-2), thus disrupting the physiological storage of DA. A reduction in VMAT-2 gene expression and protein levels in DA neurons occurs in both METH models and schizophrenic patients, marking quite impressively the overlap between these disorders [150,157,158]. It is worth mentioning that freely diffusible intra-cytosolic DA can readily undergo auto-oxidation and produce a cascade of oxidative-related damage, which is bound to the neurotoxic effects of high doses of METH [125,159,160,161]. In fact, DA auto-oxidation generates several toxic and highly reactive species such as DA-quinones, hydrogen peroxide, and superoxide radicals. Beyond METH, redox-related changes that result from an imbalance between reactive oxygen species (ROS) production and ROS clearance are implicated in schizophrenia [122]. As a result of an altered intracellular redox environment, proteins lose their native conformational fold and assume an aberrant, misfolded conformation with an abnormal tendency to aggregate into larger, often insoluble, inclusions [148,159,162]. This excessive amount of aggregate-prone proteins within DA axon terminals leads to an autophagy engulfment, which fuels a vicious cycle of oxidative stress, protein misfolding, and aggregation [125,126,148,162] (Figure 2). In fact, when administered both in vitro and in vivo, METH generates multi-lamellar whorls corresponding to stagnant autophagy vacuoles, which further develop into eosinophilic cytoplasmic inclusions within both nigral DA neurons and striatal cells [125,162,163]. These inclusions are reminiscent of PD-like Lewy bodies because they stain for typical PD markers such alpha synuclein [125,126,162,164,165]. The occurrence of analogous nigral inclusions was confirmed in human chronic METH abusers [124]. In addition, the occurrence of alpha synuclein gene (*SNCA*) polymorphisms is associated with human METH psychosis [166]. This is in line with genetic studies associating psychotic symptoms with *SNCA* multiplications [167]. As both DA neurotransmission and handling of misfolded proteins are directly bound to autophagy, it is likely that autophagy alterations represent a causative mechanism underling ongoing synaptic pathology, which could predispose to neurodegeneration. In the light of these findings, in the next paragraph, we provide evidence that ultrastructural changes related to autophagy alterations occur in schizophrenia as well.

## 5. Cytoskeletal Abnormalities and Neuronal Inclusions in Schizophrenia

Neuropathological evidence showing cytoarchitectural abnormalities and neuronal inclusions in schizophrenic patients date back to the late 1990s from post-mortem studies. In detail, ultrastructural alterations encompassing reduction and swelling of DA terminals, mitochondrial alterations, and multi-lamellar structures were reported within DA terminals of the SNpc [168]. Interestingly, these ultrastructural abnormalities are highly reminiscent of those induced by METH. In addition, cytoskeletal derangement appears as a prominent feature of the ultrastructural pathology of schizophrenia [169]. Cytoskeleton organization and dynamics depend on the fine control of microtubule assembly, which relies on the interaction of microtubules with a specific class of proteins known as microtubule-associated protein (MAP). These proteins represent a sort of cytoskeletal regulatory elements that bind microtubules to ensure their stability and integrity. Among various identified MAPs, microtubule-associated protein 2 (MAP2), which belongs to the MAP2/Tau family, is enriched in the brain and especially in dendrites, where it contributes to microtubule stabilization and overall dendritic architecture. Alterations in MAP2 immunoreactivity within the subiculum, entorhinal cortex, hippocampus, and prefrontal cortex have been suggested as the primary array of cytoskeletal abnormalities, which in turn result in impaired neurotransmission observed in schizophrenia [170,171,172,173]. Notably, a marked reduction in MAP2 immunoreactivity, along with a decrease in dendritic arbor, is reported in the primary auditory cortex (BA41) of schizophrenic subjects compared with healthy controls [174]. These structural abnormalities observed in the post-mortem auditory cortex of schizophrenic individuals may underlie altered auditory information processing, which in turn may manifest as auditory hallucinations. Moreover, pathological deposition of hyperphosphorylated MAP-tau (MAPT), which is the hallmark of several neurodegenerative disorders such as AD and frontotemporal dementia (FTD), has been described in elderly subjects with schizophrenia [175,176,177]. However, this issue is still under debate, because some autopsy studies do not report any significant difference in the prevalence of AD pathology between elderly schizophrenic patients and age-matched healthy controls [178,179,180,181]. Momeni and colleagues (2010) recently reported two relatives with an early age at onset (27 and 29 years) of schizophrenic symptoms showing a marked neuronal tau deposition, as confirmed at pathological examination [182]. Remarkably, this familial behavioral variant of frontotemporal lobar degeneration (FTLD) was associated with a novel exon 12 mutation in the conserved microtubule binding region of microtubule-associated protein tau (*MAPT*) gene, thus suggesting that disturbances in proteins involved in regulation of microtubule stability and overall cytoskeletal dynamics may accelerate tau deposition, leading to early disease onset. Notably, post-mortem analysis performed in the prefrontal cortex of schizophrenic patients revealed oligodendrocyte ultrastructural abnormalities [183]. Similarly, cytoskeletal derangements within nigro-striatal DA neurons and axons were recently evidenced in another cohort of schizophrenic brains [184]. Remarkably, a very recent neuropathological examination provided evidence for TDP-43-positive cytosolic inclusions and dystrophic neurites in the brain of a patient diagnosed with FTLD presenting brief psychotic episodes and catatonia, which is a syndrome related to schizophrenia [120]. Preliminary in vitro studies demonstrated that two proteins, namely DISC1 and dysbindin-1, which are encoded by two susceptibility genes for schizophrenia, can form insoluble protein aggregates that are reminiscent of those occurring in neurodegenerative disorders [185,186,187,188]. Intriguingly, the interactome analysis of both DISC1, dysbindin-1, and CRMP2, which is another susceptibility gene for schizophrenia, revealed common protein interactions with microtubules, actin cytoskeleton, and proteins involved in intracellular transport [189,190]. In particular, CRMP2 is a cytosolic protein enriched in the CNS, which has been implicated in microtubule stabilization, and thus in the regulation of cytoskeletal dynamics and vesicle trafficking. Remarkably, multiple proteomic studies of postmortem brains show altered CRMP2 protein levels in schizophrenic patients compared with healthy subjects [191,192,193,194]. These findings suggest that all these schizophrenia risk genes may encompass cytoskeletal stability and organization. It is worth mentioning that the reciprocal modulation between cytoskeleton dynamics and autophagy is emerging as a crucial point for neuronal homeostasis. Inhibition of the physiological microtubule transport is known to associate with an impaired shuttling of protein aggregates towards autophagy vacuoles, as well as impaired intracellular vesicle trafficking. Such an effect contributes to dysfunction in both the autophagy and secretory pathway leading to altered transmission of axonal information to and from the somato-dendritic domain [195]. The biological implication behind an impairment of microtubule dynamics is confirmed in post-mortem schizophrenic brain samples, as well as in mouse models of schizophrenia, where mTOR-dependent autophagy dysfunction is accompanied by an altered gene expression and protein levels of the microtubule-associated protein 6 (MAP6) [196,197]. These findings suggest that a close interplay between cytoskeletal dynamics and mTOR signaling is key in early axonal transport defects and altered synaptic transmission, a common pathological hallmark in schizophrenia [144,198,199,200,201].

## 6. mTOR Modulation of Dopamine Transmission in Methamphetamine and Schizophrenia

**Figure 3:**
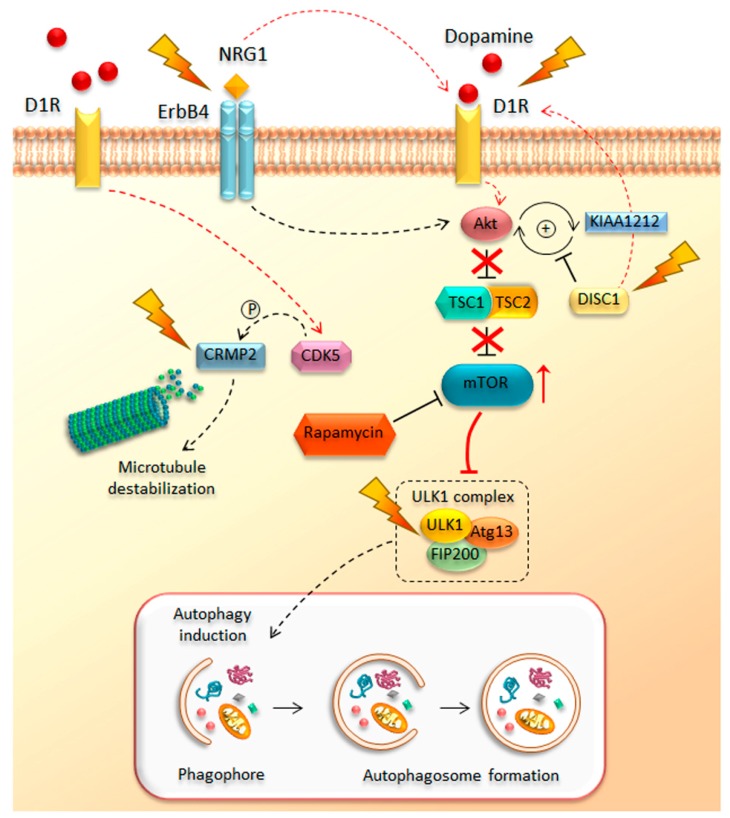
The Akt/mTOR pathway in schizophrenia. The cartoon summarizes key proteins involved in schizophrenia (lightning bolts), which converge on the overactivation of the Akt/mTOR pathway. These include disrupted in schizophrenia 1 (DISC1), neuregulin-1 (NRG1)/avian erythroblastosis oncogene B4-like protein (ErbB4), and collapsin response mediator protein 2 (CRMP2), as well as dopamine D1 receptors (D1R), which in turn are modulated by DISC1 and NRG1/ErbB4.

Despite some epidemiological evidence concerning risk factors for schizophrenia, which represents a chronic debilitating condition, the identification of the molecular mechanisms underlying its pathogenesis is still challenging. Interestingly, as has emerged from review of the literature published in the last few years, a novel scenario begins to delineate in which a dysfunctional mTOR pathway may be a key mechanism in the chain of events for the development of schizophrenia (Figure 3). In line with this, a number of genetic studies linked mTOR-related genetic alterations to schizophrenia. This is the case of the disrupted in schizophrenia 1 (*DISC1*) gene, a schizophrenia-related gene, originally discovered in a large Scottish family with a high incidence of psychiatric symptoms [202]. This gene encodes for the DISC1 ubiquitous protein, which is implicated in neurogenesis, neuronal migration, axon/dendrite, and synapse formation [203,204,205,206]. Remarkably, DISC1 plays a key role in DA neurotransmission [207]. In line with this, experimental models of DISC1 deficiency treated with METH show a significant potentiation of DA release, along with increased expression of D1R in the ventral striatum when compared with controls [208]. Mutations of DISC1 in the striatum associate with increased METH-induced behavioral sensitization suggesting that DISC1 represents a hub underlying alterations in those DA-dependent molecular mechanisms that modulate reward and sensitization in both drug abuse and mental disorders [209]. In addition, these findings strongly suggest that DISC1 alterations may increase the risk of schizophrenia by dysregulating DA release. In support of this view, DISC1 alterations are associated with pathological stress and converge in producing early alterations in DA neurotransmission during adolescence. This is a critical life-time for the development of schizophrenia [210,211]. Noteworthy, both DISC1 deficiency and over-stimulated D1Rs up-regulate the Akt–mTOR pathway [133,153,212], which highlights the impressive overlap between pathways that modulate DA neurotransmission and cell clearing systems. In particular, DISC1 acts by blocking KIAA1212, an Akt-binding partner, which directly interacts with Akt and strengthens the activation of this kinase, which represents a major mediator of the mTOR pathway. Therefore, the binding between DISC1 and KIAA1212 prevents KIAA1212-dependent Akt activation (Figure 3). This decreases Akt activity, which in turn dampens mTOR signaling [212]. Therefore, disruption of DISC1 activity, due to genetic rearrangements (i.e., balanced (1;11) (q42;q14) chromosomal translocation) or missense mutations, produces schizophrenic-like behavior, which is bound to enhanced Akt activity, over-activation of mTOR signaling, and depressed autophagy [213,214,215]. Likewise, administration of either D1R agonists or METH enhances Akt activity and over-activates mTOR signaling [133,137,216]. Remarkably, inhibition of mTOR with rapamycin reverses the effects occurring in both DISC1-shRNA and METH-treated mice, while ameliorating behavioral alterations [137,153,212]. Again, an impaired Akt signaling, achieved by neuronal deletion of rictor, a key regulatory subunit of mTORC2, contributes to schizophrenia-like phenotypes in rictor-null (KO) mice [217]. Dysregulation in Akt signaling and altered Akt protein levels were found in the frontal cortex and hippocampus of post-mortem brain samples from individuals affected by schizophrenia [218]. Since the first report by Emamian et al. (2004) [218], numerous subsequent studies further confirmed the genetic association of *AKT1* gene variants with schizophrenia, supporting the key role of impaired Akt–mTOR signaling in the pathogenesis of this psychiatric disorder [219,220,221,222,223,224]. In line with this, increased *AKT1* gene expression, due to increased hypomethylation of *AKT1* gene promoter, was detected in human METH abusers [225].

Furthermore, genetic linkage and association studies led to the identification of two additional susceptibility factors related to schizophrenia, such as neuregulin-1 (*NRG1*) and its receptor *ErbB4* [226,227]. NRG1 is a family of trophic factors that is synthesized as trans-membrane proteins displaying an extracellular epidermal growth factor (EGF)-like domain that is essential for ErbB4 receptor binding. Upon proteolytic processing, the soluble N-terminal moieties that contain the EGF-like domain is released and it acts by stimulating the ErbB4 receptor. Alterations in the NRG1/ErbB4 signaling are reported in schizophrenic brains [228]. In particular, NRG1, which is mostly involved in regulating neurodevelopment and neurotransmission, acts by binding ErbB4, a type I transmembrane receptor tyrosine kinase belonging to the family of ErbB proteins, which contain a binding site for PI3K kinase, an AKT upstream effector, in the C-terminal cytoplasmic tail (CYT) [229]. The binding between ErbB4 and PI3K activates this latter kinase, which in turn can phosphorylate and activate its downstream target Akt. Thus, changes in NRG1/ErbB4 signaling leads to a cascade of events that culminate in a dysregulation of the Akt–mTOR pathway (Figure 3). Moreover, it has been demonstrated that NRG1 also regulates DISC1 expression [230], thus further worsening the aberrancy of the Akt–mTOR pathway and the pathogenesis of schizophrenia and related behaviors. Noteworthy, NRG1/ErbB4 signaling plays a key role in DA-related behaviors by increasing DA release within the hippocampus, striatum, and prefrontal cortex [231]. In mice harboring mutated ErbB4, D1R’s levels and binding activity are significantly increased [232], suggesting that ErbB4 may be another putative protein linking increased DA activity to increased mTOR activity and depressed autophagy.

Another identified susceptibility gene for schizophrenia is the dihydropyrimidinase-like 2 (*DPYSL2*) gene. This gene is located on chromosome 8p21 and it encodes the cytosolic microtubule-associated protein CRMP2 (collapsin response mediator protein-2), which is highly expressed in the CNS and plays a role in axonal growth [233]. Several *DPYSL2* single-nucleotide polymorphisms (SNPs) have been associated with the development to schizophrenia [234,235]. Although in mammals there are three DPYSL2 transcripts (i.e., DPYSL2A, DPYSL2B, and DPYSL2C), which differ in their first exon sequence, most of the studies have focused on the DPYSL2B transcript, also known as “short” transcript [236]. Multiple functional sequence variants were identified both in and around *DPYSL2B*, including three single-nucleotide polymorphisms (SNPs) in the proximal promoter and two SNPs in intron 1, which were significantly associated with schizophrenia [236]. In vitro functional luciferase assays in both neuronal (mouse primary cortical neurons) and non-neuronal (HEK293) cell types demonstrated that in the presence of increasing concentrations of rapamycin, a polymorphic di-nucleotide repeat (DNR) in the 5′-UTR of the *DPYSL2B* gene dose-dependently decreases allele expression at translation level, suggesting a functional link between this schizophrenia high-risk allele (13 DNRs) and mTOR signaling [236]. The relationship between the *DPYSL2* gene and susceptibility to schizophrenia was recently confirmed in vivo in rats exposed to prenatal stress (PNS), which indeed is frequently reported as an environmental risk factor for developing schizophrenia in adults [237]. Remarkably, immunohistochemical and Western blot analyses performed in the prefrontal cortex and hippocampus revealed a decreased DPYSL2 expression in the PNS group compared with non-stressed control offspring [238]. CRMP2 protein levels are significantly altered by METH administration [239]. In turn, CRMP2-KO mice show altered levels of proteins and genes involved in GABA-, glutamate-, and neurotrophin-signaling pathways, which are related to both schizophrenia and METH-induced sensitization [240]. Once again, such an overlap between altered molecular mechanisms occurring in both schizophrenia and METH may be key to decipher those early events linking CRMP2 and DA activity. In line with this, the dendritic spine-regulating activity of CRMP2 is under the control of the cyclin-dependent kinase 5 (CDK5) [241]. CDK5 is a key second messenger participating in METH-induced behavioral sensitization [130,242,243]. Both administration of amphetamines and stimulation of D1R induce a significant increase of *CDK5* gene expression and protein levels, which, at molecular level, associates with increased dendritic spine density and hyper-phosphorylation of the cytoskeletal tau protein [244,245,246]. In detail, the activation of CDK5 by D1R occurs via proteolysis of p35, the binding partner of CDK5. Remarkably, the marked reduction of p35 levels in schizophrenic brains, which mirrors enhanced CDK5 activity [247], suggests a role for CDK5-CRMP2-dependent alterations of cytoskeleton architecture and psychiatric behavior.

## 7. A Step Forward about a Role of Autophagy in the Pathophysiology of Schizophrenia

While recent advances in molecular psychiatry have identified several mTOR-related schizophrenia risk genes, the role of autophagy in schizophrenia has been recently investigated. Remarkably, the identification of rare genetic variants of *ULK1* in a cohort of schizophrenic patients by means of exome sequence analysis strengthens the idea of a key role of both disrupted mTOR signaling and autophagy in the pathophysiology and susceptibility to schizophrenia [248] (Figure 3). The first evidence of a dysregulation of autophagy in schizophrenia was provided in 2011 by the Horesh group, who performed gene expression profile analysis in different brain areas of post-mortem schizophrenic patients compared with healthy controls, with no evidence of concomitant dementia [249]. The study revealed profound differences between the two groups, especially when looking at Broadman area 22 (BA 22), which is associated with positive symptoms, mainly auditory-verbal hallucinations or “hearing voices” [250,251]. In particular, at BA 22, the vast majority of abnormally expressed genes referred to key autophagy genes (i.e., *BECN1*, *ULK2*, *ATG3*), which were significantly down-regulated compared with controls [249]. A few months later, another transcriptomic study reported a BA 22-specific down-regulation in several autophagy-related genes, thus strengthening the link between impaired autophagy and schizophrenia positive symptoms [252]. Furthermore, the transcriptional analysis performed on the very same post-mortem samples demonstrated no substantial changes in the mRNA levels of the above-mentioned autophagy-related genes within the anterior prefrontal cortex (BA 10), which is mainly involved in schizophrenic negative symptoms and cognitive dysfunction, thus reinforcing the involvement of an impaired autophagy in mediating positive symptoms. Later on, further analysis reported a disruption of the autophagy pathway also in the hippocampus of post-mortem schizophrenic patients [197]. In detail, the analysis of mRNA expression of a key protein for autophagy initiation, namely beclin1, revealed a significant region-specific reduction in hippocampal samples from 12 schizophrenic patients compared with 12 age-matched healthy controls. The deficiency in hippocampal beclin1 transcript levels matches those observed in haploinsufficient mice for the activity-dependent neuroprotective protein (ADNP) (ADNP^+/−^ mice), a transgenic model of schizophrenia [197]. ADNP is an essential protein for brain development and it has been shown to physically interact with a key protein in autophagosome biogenesis and maturation, namely LC3 [253]. Remarkably, a co-immunoprecipitation assay performed in a hippocampal protein fraction from ADNP^+/−^ mice showed a dramatic reduction in the ADNP–LC3 protein interaction, which correlates with decreased ADNP expression [197]. A reduction of ADNP and its homologous protein, ADNP2, is observed in schizophrenic patients [254], and it is recapitulated in Map6-deficient (Map6^+/−^) mice, another transgenic model of schizophrenia [196]. Immuno-histochemical analysis showed a three-fold decrease in the number of beclin1-positive cells in Map6^+/−^ mice. These results were confirmed at transcriptional level by demonstrating a reduced expression of BECN1 mRNA. On the other hand, chronic treatment with an eight-amino-acid peptide snippet from ADNP (NAP), also known as davunetide, restored both Beclin1 and ADNP mRNA levels along with ADNP-LC3 interaction, thus providing neuroprotection while ameliorating schizophrenic-like behavioral and cognitive deficits in Map6^+/−^ mice [196]. A recent phase II, multicenter, double-blind, randomized clinical trial has shown an improvement in cognitive performance of schizophrenic patients treated with NAP (AL-108; 5 and 30 mg/day, intranasally) versus placebo-treated patients [255]. These pieces of evidence corroborate findings showing that several autophagy inducers, such as lithium, rapamycin, and Food and Drug Administration (FDA) approved antipsychotic drugs are effective to treat psychosis including schizophrenia [59,60,61,62,109,256,257,258,259,260]. Notably, high-throughput image-based screens performed by Zhang et al. (2007) [60] on a human glioblastoma H4 cell line expressing human LC3 coupled with green fluorescent protein (GFP) led us to disclose that three typical antipsychotic drugs (fluspirilene, trifluoperazine, and pimozide) are effective autophagy inducers. In particular, pimozide provides an mTOR-independent autophagy induction, because it directly activates AMPK1, which in turn promotes autophagy through the phosphorylation of ULK1 [260]. In contrast, chlorpromazine, which is a typical antipsychotic agent, induces autophagy by inhibiting the Akt/mTOR pathway [59]. Recently, in vitro studies on the effects of second-generation, atypical antipsychotics demonstrated that sertindole and clozapine are potent autophagy inducers in both neuronal and non-neuronal cell lines [257,261]. Similar to pimozide, clozapine activates the autophagy process via the AMPK–ULK1–Beclin1 pathway, as evidenced by increased levels of autophagy markers (i.e., LC3-II and Atg5–Atg12 conjugate); increased phosphorylation of AMPK and its downstream substrates, namely ULK1 and beclin1; and an increased number of autophagosomes in the frontal cortex in clozapine-treated rats [259]. Most reports evidenced autophagy induction by neuroleptics indirectly, only by measuring the degradation of autophagy-dependent substrates. For instance, the increase in autophagy flux induced by pimozide occurs along with a depression of phosphorylated tau in a transgenic mouse model of AD [260]. Again, the effects of two typical antipsychotics, trifluoperazine and haloperidol, on autophagy have been demonstrated indirectly [262,263]. For instance, haloperidol occludes huntingtin aggregation [262]. Chronic clozapine treatment (20 mg/kg/day) reduces Aβ deposition [264]. Although typical and atypical antipsychotics may alleviate diseases featuring aberrant protein misfolding and accumulation, in vivo systematic investigations regarding the efficacy and the molecular mechanisms of these drugs on autophagy have been questioned [265]. This issue is biased by the routine intake of neuroleptics by most schizophrenic patients for long time intervals, sometimes lasting decades.

## 8. Conclusions and Future Perspectives

The exponential development in the past few years of genome-wide linkage studies and high-throughput genotyping technologies has led to the identification of many other susceptibility genes for schizophrenia, and this list is expected to grow further. However, the molecular mechanisms underlying schizophrenia are far from being deciphered. Up-to-date neuropathological studies performed on post-mortem schizophrenic brains appear to be scattered and they have not yielded to the identification of a distinct neuropathological hallmark. This is due to the limited sample availability and confounding interpretation of pathological data when comparing antipsychotic-treated and non-treated patients. This contrasts with classic neurodegenerative disorders where the accumulation of misfolded or aggregated proteins within neurons may imply a dysregulation of autophagy. Again, most experimental models available so far fail at large to reproduce most features of schizophrenia. Thus, methamphetamine remains an appropriate model compared with genetic manipulation to decipher the molecular progression underlying the pathophysiology of schizophrenia. In fact, METH bridges autophagy alterations with altered DA transmission and degeneration that is reminiscent of schizophrenia.

The present manuscript reviewed genetic and biochemical evidence that suggests that autophagy impairment may be involved in early DA neurotransmission, leading to synaptic dysfunction, which underlies some psychiatric disorders. An ongoing and persistent autophagy dysfunction that occludes handling of misfolded proteins while fueling synaptic alterations predisposes to the onset of degeneration. This scenario, depicting schizophrenia as an autophagy-dependent progressive synaptic pathology, may be a ground for planning the use of mTOR inhibitors and autophagy inducers as early treatment intervention.

## Figures and Tables

**Table 1:** Altered proteins converging on the mammalian target of rapamycin (mTOR) pathway during schizophrenia and methamphetamine addiction. DISC1—disrupted in schizophrenia 1; Akt—protein kinase B; NGR1—neuregulin-1; ErbB4—avian erythroblastosis oncogene B4-like protein; CRMP2—collapsin response mediator protein 2; CDK5—cyclin-dependent kinase 5.

Protein	Schizophrenia	Methamphetamine
DISC1	[153,212,213,214,215]	[208,209]
Akt	[218,219,220,221,222,223,224]	[225]
NRG1/ErbB4	[228,230,232]	[231]
CRMP2	[236,238]	[239,240]
CDK5/p35	[247]	[130,242,243]

## Abbreviations

AD	Alzheimer’s disease
ADNP	activity-dependent neuroprotective protein
Akt	protein kinase B
ALS	Amyotrophic lateral sclerosis
Aβ	amyloid-β
BA 22	Broadman area 22
BA41	Broadman area 41
CDK5	cyclin-dependent kinase 5
CMA	chaperone-mediated autophagy
CNS	central nervous system
CRMP2	collapsin response mediator protein 2
D1R	dopamine receptor type 1
DA	dopamine/dopaminergic
DAT	dopamine transporter
DISC1	disrupted in schizophrenia 1
DLB	Dementia with Lewy bodies
DNR	di-nucleotide repeat
DPYSL2	dihydropyrimidinase-like 2
ErbB4	avian erythroblastosis oncogene B4-like protein
FRKBP12	FK506-binding protein 12
FTD	Frontotemporal dementia
FTLD	Frontotemporal lobar degeneration
GFP	green fluorescent protein
GPCRs	G-protein coupled receptors
HD	Huntington’s disease
MAP	microtubule-associated protein
MAPT	microtubule-associated protein tau
METH	methamphetamine
mTOR	mammalian Target Of Rapamycin
mTORC1	mammalian Target Of Rapamycin complex 1
mTORC2	mammalian Target Of Rapamycin complex 2
NDDs	neurodegenerative disorders
NFT	neurofibrillary tangles
NRG1	neuregulin-1
PD	Parkinson’s disease
PI3k	phosphatidylinositol-3-Kinase
PIP2	phosphatidylinositol-4,5-phosphate
PIP3	phosphatidylinositol-3,4,5-phosphate
PNS	prenatal stress
ROS	reactive oxygen species
RTKs	receptor tyrosine kinase receptors
SNARE	Soluble NSF (N-ethylmaleimide-sensitive factor) attachment protein receptor
SNCA	alpha synuclein gene
SNpc	substantia nigra pars compacta
SNPs	single-nucleotide polymorphisms
SOD1	superoxide dismutase 1
TDP-43	TAR DNA-binding protein 43
TEM	transmission electron microscopy
TORC	target of rapamycin complex
TSC	tuberous sclerosis complex
TSC1	hamartin
TSC2	tuberin
VMAT-2	vesicular monoamine transporter type 2
